# Research on a Dual-Mode Infrared Liquid-Crystal Device for Simultaneous Electrically Adjusted Filtering and Zooming

**DOI:** 10.3390/mi10020137

**Published:** 2019-02-19

**Authors:** Zhonglun Liu, Mingce Chen, Zhaowei Xin, Wanwan Dai, Xinjie Han, Xinyu Zhang, Haiwei Wang, Changsheng Xie

**Affiliations:** 1 China-EU Institute for Clean and Renewable Energy, Huazhong University of Science & Technology, Wuhan 430074, China; liuzhonglun@hust.edu.cn; 2 National Key Laboratory of Science and Technology on Multispectral Information Processing, Huazhong University of Science & Technology, Wuhan 430074, China; mclovelz@hust.edu.cn (M.C.); D201577548@hust.edu.cn (Z.X.); M201672381@hust.edu.cn (W.D.); M201672398@hust.edu.cn (X.H.); 3 School of Automation, Huazhong University of Science & Technology, Wuhan 430074, China; 4 Wuhan National Laboratory for Optoelectronics, Huazhong University of Science & Technology, Wuhan 430074, China; hiway@hust.edu.cn (H.W.); Cs_xie@hust.edu.cn (C.X.)

**Keywords:** dual-mode liquid-crystal (LC) device, infrared Fabry–Perot (FP) filtering, LC micro-lenses controlled electrically

## Abstract

A new dual-mode liquid-crystal (LC) micro-device constructed by incorporating a Fabry–Perot (FP) cavity and an arrayed LC micro-lens for performing simultaneous electrically adjusted filtering and zooming in infrared wavelength range is presented in this paper. The main micro-structure is a micro-cavity consisting of two parallel zinc selenide (ZnSe) substrates that are pre-coated with ~20-nm aluminum (Al) layers which served as their high-reflection films and electrodes. In particular, the top electrode of the device is patterned by 44 × 38 circular micro-holes of 120 μm diameter, which also means a 44 × 38 micro-lens array. The micro-cavity with a typical depth of ~12 μm is fully filled by LC materials. The experimental results show that the spectral component with needed frequency or wavelength can be selected effectively from incident micro-beams, and both the transmission spectrum and the point spread function can be adjusted simultaneously by simply varying the root-mean-square value of the signal voltage applied, so as to demonstrate a closely correlated feature of filtering and zooming. In addition, the maximum transmittance is already up to ~20% according the peak-to-valley value of the spectral transmittance curves, which exhibits nearly twice the increment compared with that of the ordinary LC-FP filtering without micro-lenses.

## 1. Introduction

In recent years, the infrared detection technology [1] has been developed rapidly, because it presents several advantages including relatively long working distance, better anti-interference, full-time availability, and strong penetration of haze smoke or dust. So far, it has played a significant role in many applications such as the earth resources survey [2], global pollution and disaster warning [3,4,5], biomedical diagnosis [6,7], food safety monitoring [8,9,10,11,12], and so on. As demonstrated, the electromagnetic radiation out from targets exhibits its own specific spectral characters, which contain objective surface or structural information or chemical composition. Unfortunately, the spectral clues acquired by us are not only originated from targets, but also from environmental circumstances, which means that a mixed broad spectrum can be expected. So, it is of great importance to select only the needed spectral components from incoming beams.

Recently, many methods including typical Mach–Zehnder interferometer [13], Michelson interferometer [14], Bragg grating [15], electro-optical or acousto-optical tuning [16], and Fabry–Perot (FP) micro-cavity [17,18,19,20] were constructed successfully. Among them, the FP micro-cavity based on the common interference theory had been developed quickly due to its typical characteristics of ultra-small size and relatively low cost and very high spectral resolution. Combining with the micro-electro-mechanical system (MEMS) [21,22], the electrically tunable MEMS-FP filter had already been effectively constructed. According to the MEMS architecture developed, the depth of the FP micro-cavity could be altered through moving mechanical parts, which supported the mirrors to achieve the spectral choice or further spectral adjustment. At the same time, the liquid-crystal (LC)-FP filters [23,24,25,26] composed of the similar FP micro-cavity filled by LC materials had also attracted much attention. As shown, the depth of the FP micro-cavity was fixed, which indicated that the choice or adjustment of the spectral component needed could be conducted by only varying the equivalent index of refraction of the LC layer without any mechanical movement. As known, the characteristics of the lenses with variable focus, as core parts in modern imaging equipment, would greatly determine the final performances of the imaging system. So, the electrically controlled LC micro-lenses [27,28,29,30,31,32] can be a perfect type of micro-device for flexibly readjusting the patterned light-fields compressed over the surface of the sensor array by main optical system, and thus applied to advanced light-field cameras [33], autostereoscopic devices [34,35,36,37] and wave-front measurement and correction sensors [38,39,40].

In our previous work, a graphene-based LC micro-lens array with a tunable focal length had been developed successfully [41,42]. In order to develop a new kind of dual-mode LC micro-device for simultaneous electrically adjusted filtering and zooming, a structured FP cavity was incorporated into a common LC micro-lens array. The experimental results showed that the maximum transmittance of the spectral micro-beams was already up to ~20% according the peak-to-valley value of the spectral transmittance curves, which exhibited nearly twice the increment compared with the ordinary LC-FP filter. A needed zooming performance was acquired, as shown in the near infrared (NIR) band. When the root-mean-square (RMS) value of the signal voltage was varied from ~2 V_rms_ to ~16 V_rms_, the point spread function (PSF) of the micro-lenses can be adjusted efficiently, and thus the focal length altered from ~2.80 mm to ~3.93 mm.

## 2. Materials and Methods

### 2.1. Device Design and Fabrication

**Figure 1:**
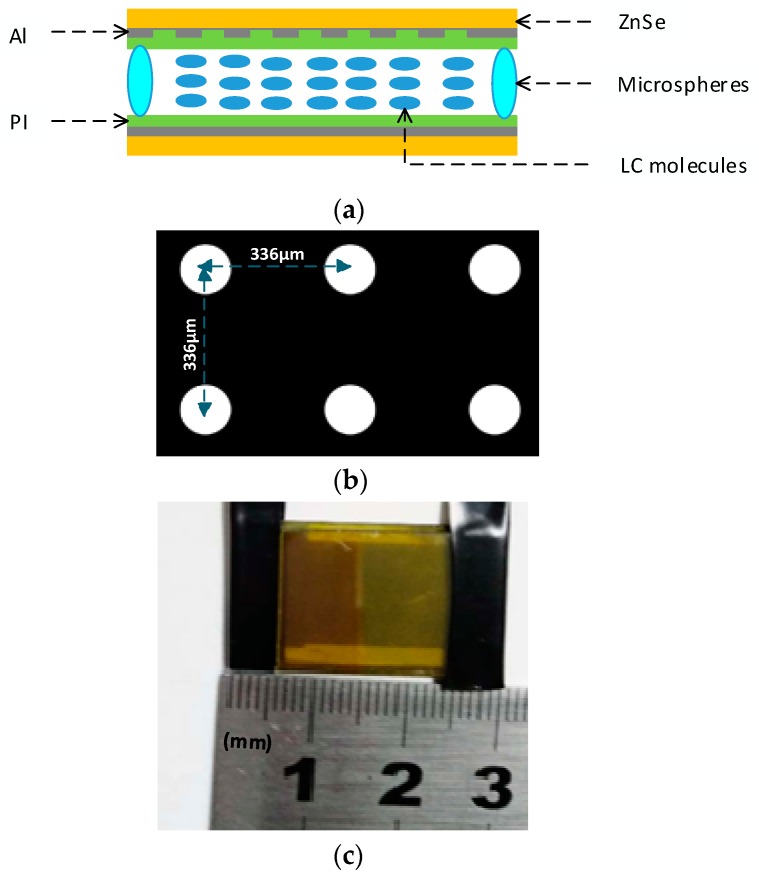
The dual-mode liquid-crystal (LC) micro-device—(**a**) main architecture; (**b**) patterned Al electrode; and (**c**) appearance of the final micro-device.

The basic micro-structure of the dual-mode LC micro-device is shown in Figure 1. Figure 1a exhibits the main architecture. Both the patterned Al electrode and the appearance of the actual micro-device are shown in Figure 1b,c, respectively. In the micro-device, ZnSe were chosen as the substrates according to the design of the transmittance being more than 70% in infrared region and having a good tolerance at a relatively high temperature for fabrication process. Firstly, a ~20-nm aluminum (Al) film as a high-reflection film and electrode was simultaneously coated over the surface of ZnSe substrate, since it presented a high reflectivity in the infrared region and a nice electrical conductivity. The top electrode of the micro-device was patterned by 44 × 38 circular micro-holes of 120 μm diameter, which also meant a 44 × 38 micro-lens array. The center-to-center distance between adjacent micro-holes was 336 μm. An alignment layer for initially directing LC molecules made of a thin film of polyamide (PI) was firstly coated on the top and bottom Al film, respectively, and then rubbed in anti-parallel direction to form V-grooves. Both ZnSe substrates were placed in parallel to each other and separated by glass micro-spheres with a typical diameter of ~12 μm so as to construct a stable micro-cavity, where a layer of nematic LC materials of Merck E44 (*n_o_* = 1.5280, *n_e_* = 1.7904, *K*_11_ = 15.5 × 10^−6^, *K*_22_ = 13.0 × 10^−6^, *K*_33_ = 28.0 × 10^−6^, *ep* = 22*ε*_0_, *et* = 5.2*ε*_0_) was fully filled. The outline size of the final micro-cavity fabricated was about 3 cm × 2 cm × 2 cm.

### 2.2. Theoretical Analysis

According to the design, the multi-interference based on FP effect was performed between the micro-holes or LC micro-lenses. Equation (1) shows a wavelength selection effect, and a relationship between the wavelength and the corresponding transmittance is also revealed in Equation (2), when the incident beams are perpendicular to the micro-device.

(1) mλ=2ndcosα

where *m* is a positive integer, *λ* is the wavelength of the beams satisfying the resonance condition, *n* is the equivalent refractive index of LC materials, *α* is the incident angle of incoming beams, and *d* is the depth of the micro-cavity.

(2) $$T\left(\lambda \right)={\left(1-\frac{A}{1-R}\right)}^{2}\frac{1}{1+\frac{4R}{{\left(1-R\right)}^{2}}\sin^{2}\left(\frac{2\pi nd}{\lambda }+\varphi \right)}$$

where *T* is the transmittance of the micro-device, *A* is the absorptivity of all materials used in fabricating the micro-device, *R* is the reflectivity of Al film, and *φ* is the phase shift of the beams.

If the incident beams are perpendicularly passing through the micro-device, *α* = 0, then only needed spectral micro-beams in a specific wavelength narrow band and satisfying the condition according to Equation (1) can emit at a high transmittance, so as to realize desired filtering operation. Generally, there are two ways to adjust the emitting wavelength—one is to vary the depth of the cavity, as shown in the MEMS-FP; the other is to alter only the refractive index of the medium between the electrodes of the micro-device, which is exactly the situation proposed by us. Since the LC ordering was influenced not only by surface anchoring but also by bulk elastic energy [43,44], a key parameter *d* was introduced to represent the micro-device and an experienced value of 12 μm was used to shape the functional LC layer for exhibiting both the zooming function of the LC micro-lens and the filtering function of the FP cavity, simultaneously.

When an electric field was stimulated effectively between the top and bottom electrodes, LC molecules distributed over the surface of the micro-hole would stay in their initial constraint state according to the appearance of the shaped V-grooves, because no electric field can be shaped at the center of each micro-hole. In other words, outside the micro-holes or in the effective FP region, the electric field was perpendicular to the electrode, and most of LC molecules were driven to align along the electric field lines except for partial LC molecules directly contacting with PI layer of the top electrode, and thus strongly directed by PI fabricated V-grooves. It should be noted that the closer to the central area of the micro-hole, the greater the distortion of the electric field lines, and the larger the inclination angle of LC molecules distributed over the middle LC layer corresponding to each LC micro-lens. In addition, the inclination can also be related to the intensity of the electric field stimulated. Generally, it would increase when the electric field was enhanced. The equivalent indices of LC materials varied with the change of the inclination angle of LC molecules, as revealed in Equation (3).

(3) $$n=\frac{n_{o}n_{e}}{\sqrt{n_{e}{}^{2}\cos^{2}\theta +n_{o}{}^{2}\sin^{2}\theta }}$$

where *θ* is the inclination angle, *n_o_* and *n_e_* are the refractive indices of LC materials corresponding to the ordinary light and the extraordinary light, respectively. It can be seen that when the stimulated electric field was enhanced, the inclination of LC molecules became larger and then the equivalent index of refraction was decreased. As a result of the reduction of the stimulated electric field, the inclination would be small and thus resulted in an increase of the equivalent index of refraction. Therefore, the refractive indices of LC materials would decrease gradually from the center with respect to the micro-hole to the edge, so as to lead to a needed decrease of light path. Eventually, the beam converging effect appears. Furthermore, since both the transmission spectrum and the focal length of the micro-device are obviously related with the equivalent index of the LC layer or the inclination of the LC molecules, they will be varied simultaneously with the alteration of the signal voltage applied.

## 3. Experiments and Results

**Figure 2:**
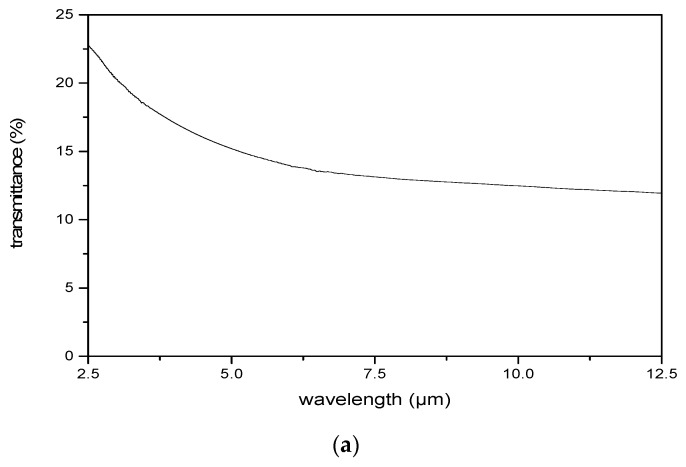

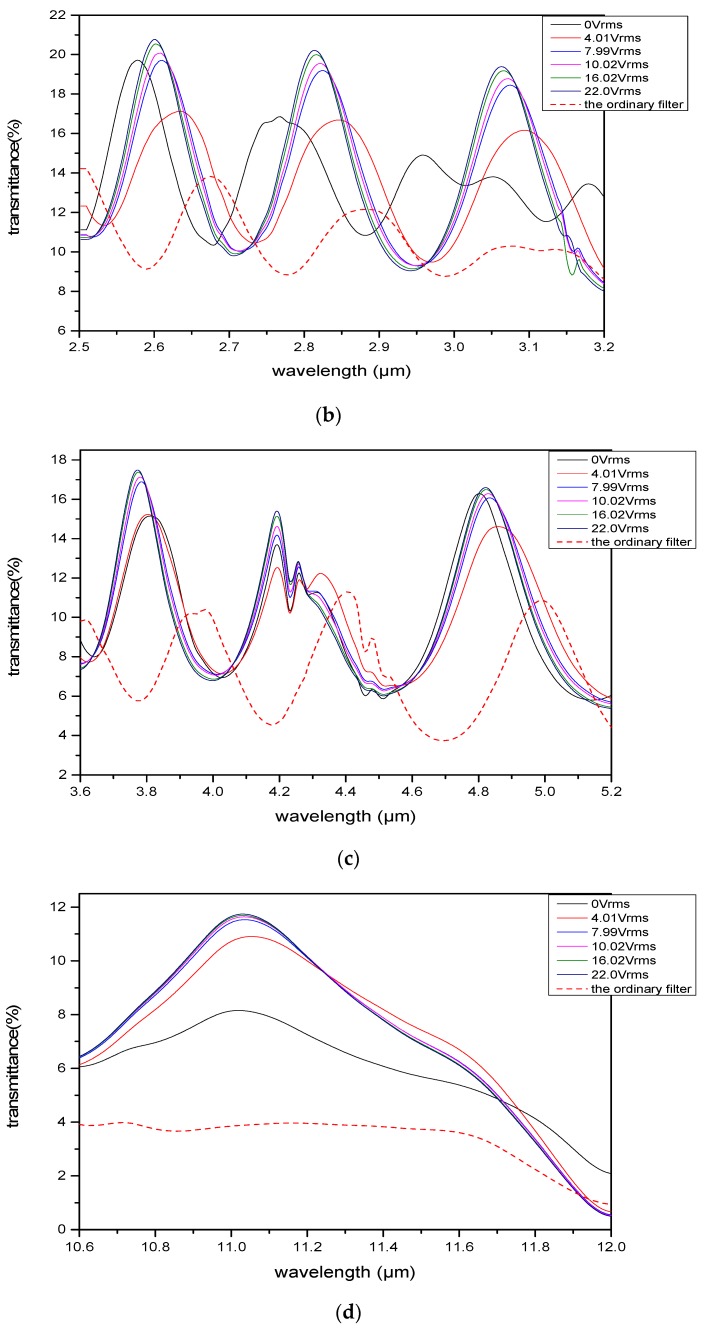
The results of spectral filtering showed the transmittance of (**a**) the substrate covered by Al film; (**b**) wavelength band from 2.5 to 3.2 μm; (**c**) wavelength band from 3.6 to 5.2 μm; and (**d**) wavelength band from 10.6 to 12.0 μm. The signal voltages included were 0 V_rms_, 4.01 V_rms_, 7.99 V_rms_, 10.02 V_rms_, 16.02 V_rms_, and 22.00 V_rms_.

During measurements, a Fourier transform infrared spectrometer (FTIR) of EQUINOX 55 (Bruker, Madison, WI, USA) was used to test the transmission spectrum. The sampling rate was about 80 spectra per second, and the wave numbers were from 4000 cm^−1^ to 400 cm^−1^, which meant that the wavelength range was from 2.5 μm to 25 μm. A square wave signal with a 1000 Hz frequency and a duty ratio of 1:1 was applied, and its voltage increased gradually from 0 V_rms_ to 22 V_rms_. Figure 2 reveals the measurement results about spectral filtering. The transmission of ZnSe substrate covered with Al film is shown in Figure 2a. Figure 2b–d show the transmittance spectra of the dual-mode LC micro-device in several wavelength bands including ~2.5 to ~3.2 μm, ~3.6 to ~5.2 μm, and ~10.5 to ~12.0 μm, at the signal voltage of 0 V_rms_, 4.01 V_rms_, 7.99 V_rms_, 10.02 V_rms_, 16.02 V_rms_, and 22.00 V_rms_.

As shown, the transmission of the substrate coated with Al film decreased from ~22.5% to ~12.5% with the increase of the wavelength from ~2.5 to ~12.5 μm, and the average value was about 15%. So, the reflectance of the micro-mirror was about 85%. The spectra indicated by solid lines were the performances of the dual-mode LC micro-device. Since LC of E44 presented a strong absorption in the wavelength range ~3.2 to ~3.6 μm, the wavelength bands of ~2.5 to ~3.2 μm and ~3.6 to ~5.2 μm were analyzed carefully. It can be clearly seen that there were three peaks in each band, and the maximum peak transmittance was up to ~20%. The light loss was mainly due to the strong absorption of Al film and ZnSe and LC materials. As shown, an effective transmission peak with a maximum value of ~12% appeared in the long-wave infrared. When the RMS value of the signal voltage was varied, the transmission spectrum was also shifted, so as to prove the electrically adjusting feature of the developed micro-device. As demonstrated, the maximum adjusting range had already extended to ~80 nm. Furthermore, the dotted lines in the pictures represent the spectrum of the ordinary LC-FP filter without micro-lenses at 0 V_rms_. Obviously, the transmittance of the micro-device proposed was around 5% higher at the same voltage. In addition, the peak-to-valley value of the obtained spectrum exhibited nearly twice the increment compared with the ordinary one, which was a new result.

**Figure 3:**
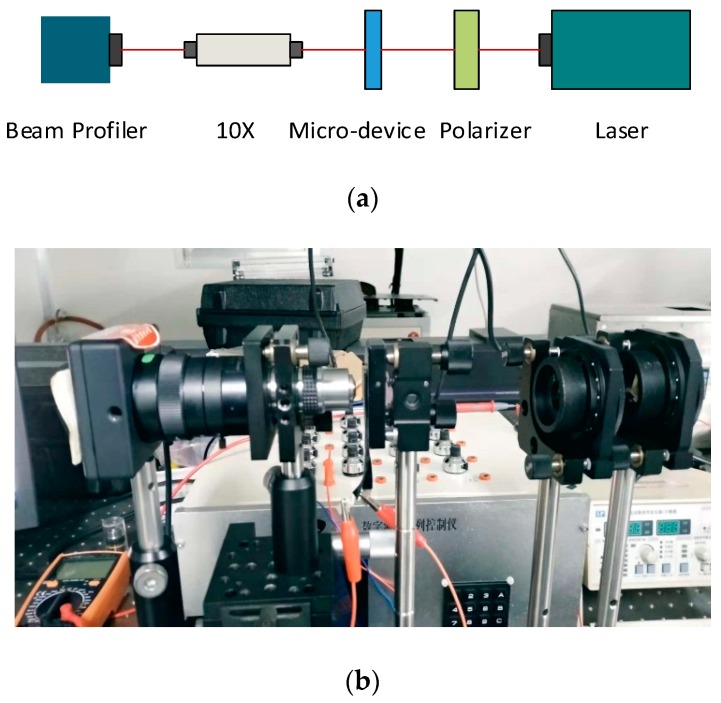
Optical measurement platform for zooming performance—(**a**) schematic diagram and (**b**) actual testing platform.

The measurement platform for acquiring zooming performances is exhibited in Figure 3. Figure 3a shows the schematic diagram of the optical path, and Figure 3b displays the actual system. During measurements, an infrared beam was emitted from a NIR laser with a central wavelength of ~980 nm, and then weakened by two polarizers, and finally a vertical polarization component being into the micro-device and continuously received by a beam profiler with a magnifying objective of 10×.

**Figure 4:**
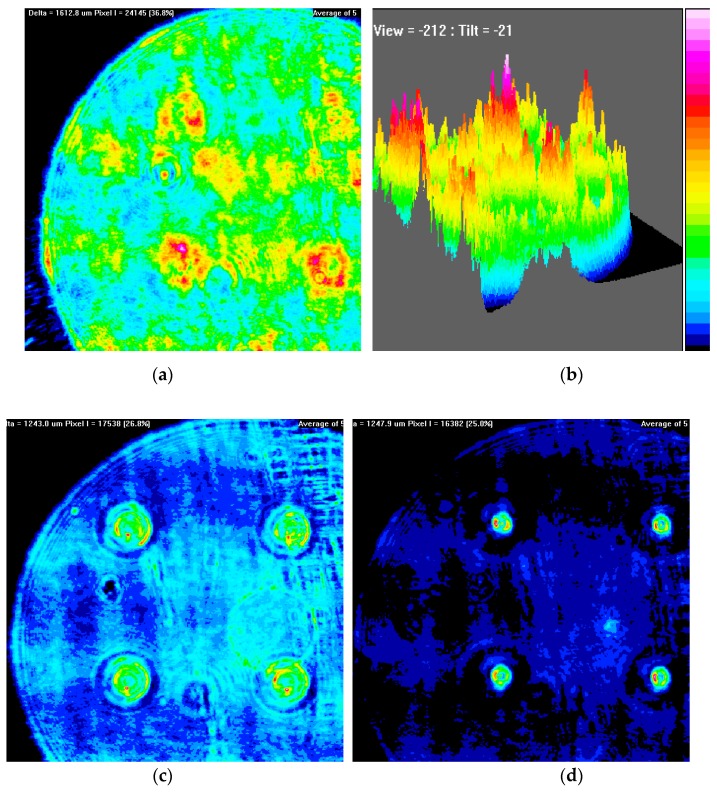

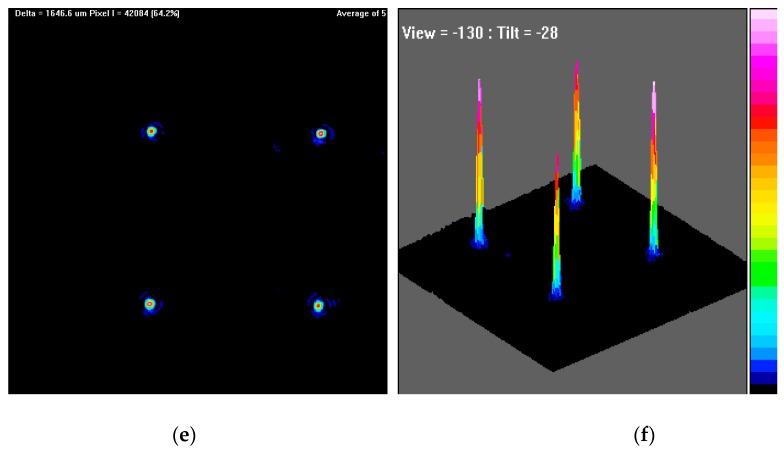
The light intensity distribution formed by LC micro-lenses. (**a**) The 2D light intensity distribution at 0 V_rms_ at the distance of ~3.550 mm and (**b**) the 3D light intensity distribution at 0 V_rms_ at the distance of ~3.550 mm. The 2D light intensity distribution at ~4 V_rms_ at the distance of (**c**) ~2.215 mm, (**d**) ~2.555 mm, and (**e**) ~3.550 mm. (**f**) The 3D light intensity distribution at ~4 V_rms_ at the distance of ~3.550 mm.

**Figure 5:**
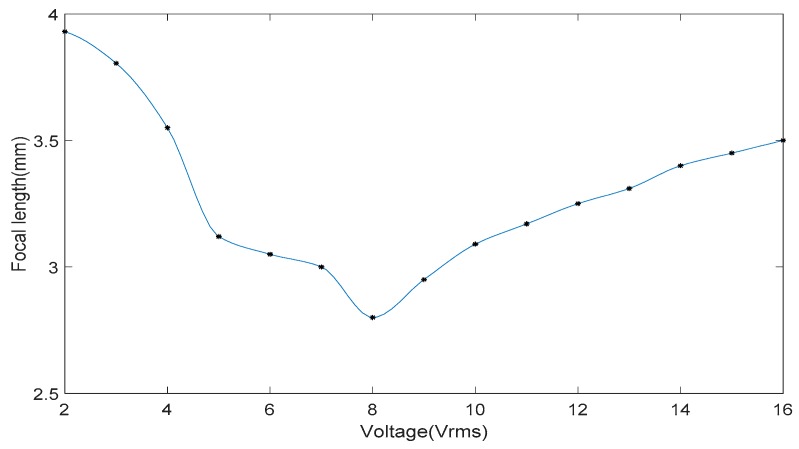
The relationship between the focal length of the dual-mode LC micro-device and the signal voltage applied.

In experiments, the applied signal voltage was kept constant, for example, at ~4 V_rms_, and then the distance between the objective lens and the center of the micro-device was adjusted continuously. Figure 4 shows the light intensity distribution formed by LC micro-lenses. The 2D and 3D light intensity distributions at 0 V_rms_ corresponding to a distance of ~3.550 mm are displayed in Figure 4a,b. Figure 4c–e exhibit the 2D light intensity distribution at ~2.215 mm, ~2.555 mm, and ~3.550 mm at ~4 V_rms_. Figure 4d displays a 3D light intensity distribution at the distance of ~3.550 mm at ~4 V_rms_. Testing results suggested that the micro-device demonstrated a needed micro-beam convergence performance when an electric field was applied, and the facula size of each LC micro-lens decreased gradually with the distance increasing from ~2.215 mm to ~3.550 mm at ~4 V_rms_. Since the PSF was already centralized and sharpened fully at ~3.550 mm, this value can be taken as the focal length of the micro-device at ~4 V_rms_. By changing the applied signal voltage and repeating the measurements, the focal length of the LC micro-device under different voltages can be acquired. The relationship between the focal length and the applied signal voltage is indicated in Figure 5. When the voltage was 2 V_rms_, the focal length was ~3.93 mm and then decreased with the increase of the voltage. As shown, the minimum value of ~2.80 mm was at 8 V_rms_ and then increased gradually to ~3.50 mm at ~16 V_rms_. Beyond this working range, the focusing performance was poor because the electric field in the LC micro-lenses was not suitable for forming an effective gradient distribution and variance of the refractive index.

**Figure 6:**
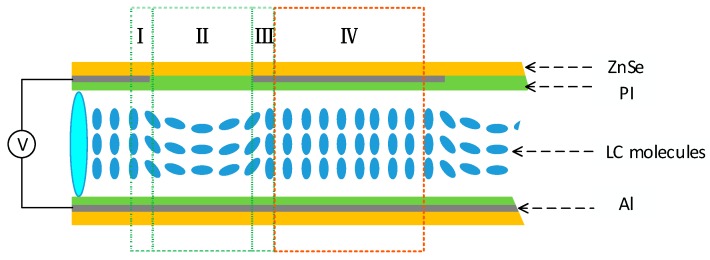
The cross-sectional view of the structural piece of the dual-mode LC micro-device.

Figure 6 shows a cross-sectional view of the structural piece of the dual-mode LC micro-device. The working area of each LC micro-lens includes region -I, -II, and -III, and the width of the region -I or -III, as shown, is ~20% of that of region -II or the diameter of a micro-hole. Moreover, when the light is incident upon the surfaces of the region -I and -III, the emitted beams should be in a narrow wavelength band, due to the micro-beam filtering effect of the FP cavity constructed, and finally resulted in a slight weakness of the micro-beam intensity converged by the LC micro-lens. For the FP cavity, the main working area is region -IV, and considering the superposition of light going through region -I, -II, and -III, the final spectral transmittance will be increased, as shown in Figure 2.

From the experiments, it was clear that the proposed micro-device had some special features derived from a FP filter and an arrayed LC micro-lens, because the filtering and zooming operation were closely correlated. When varying the RMS value of the signal voltage applied, the transmission spectrum and focal length of the micro-device were adjusted simultaneously, so as to demonstrate a featured spectral function of the dual-mode micro-device different from common FP filters and LC micro-lenses coupled with sensors. In addition, the obtained infrared spectrum involved several small wavelength bands. The focusing beams shaped at the sensor array also showed a relatively narrow wavelength region corresponding to incident infrared radiation. Through changing the applied electric filed over the functional LC layer, the filtered wave bands were varied. Then, the wave bands of focusing beams at the surface of the sensor array were also affected by changing the focused spectral components, which also involved micro-beams from the region I and III, so as to demonstrate a complicated behavior of adjusting spectrum. Currently, the relationship between filtered wave bands and focused wave bands is being studied carefully.

## 4. Conclusions

In this paper, a new kind of dual-mode LC micro-device for simultaneously electrically controlled filtering and zooming in infrared band was proposed. The testing results showed that the spectral component with needed light frequency or wavelength band can be effectively selected from incident micro-beams, and the transmission spectrum and the zooming performance were able to be adjusted simultaneously by varying only the RMS value of the signal voltage applied over the micro-device. The correlation relationship between the filtered wave bands and the focused wave bands should be discussed carefully. Due to the ability of recording the light field information of targets by LC micro-lenses, the dual-mode micro-device has a good application prospect in integrating it into infrared light field cameras for conveniently conducting digital refocusing and three-dimensional pattern reconstructing.
